# Who started first? Bird species visiting novel birdfeeders

**DOI:** 10.1038/srep11858

**Published:** 2015-07-07

**Authors:** Piotr Tryjanowski, Federico Morelli, Piotr Skórka, Artur Goławski, Piotr Indykiewicz, Anders Pape Møller, Cezary Mitrus, Dariusz Wysocki, Piotr Zduniak

**Affiliations:** 1 Institute of Zoology, Poznań University of Life Sciences, Wojska Polskiego 71C, PL-60-625 Poznań, Poland; 2 Faculty of Biological Sciences, University of Zielona Góra, Prof. Z. Szafrana St. 1, PL-65-516 Zielona Góra, Poland; 3 Czech University of Life Sciences Prague, Faculty of Environmental Sciences, Department of Applied Geoinformatics and Spatial Planning, Kamýcká 129, CZ-165 00 Prague 6, Czech Republic; 4 Institute of Nature Conservation, Polish Academy of Sciences, Mickiewicza 33, PL-31-120 Kraków, Poland; 5 Siedlce University of Natural Sciences and Humanities, Faculty of Natural Science, Department of Zoology, Prusa 12, 08–110 Siedlce, Poland; 6 Department of Zoology and Landscaping, University of Science and Technology, Ks. A. Kordeckiego 20, PL-85-225 Bydgoszcz, Poland; 7 Laboratoire d’Ecologie, Systématique et Evolution, CNRS UMR 8079, Université Paris-Sud, Bâtiment 362, F-91405 Orsay Cedex, France; 8 Department of Zoology, University of Rzeszów, Zelwerowicza 4, PL-35-601 Rzeszów, Poland; 9 Department of Department of Vertebrate Zoology and Anthropology, University of Szczecin, Wąska 13, PL-71-412 Szczecin, Poland; 10 Department of Avian Biology & Ecology, Faculty of Biology, Adam Mickiewicz University, Umultowska 89, PL-61-614 Poznań, Poland

## Abstract

Adapting to exploit new food sources may be essential, particularly in winter, when the impact of food limitation on survival of individuals is critical. One of the most important additional sources of food for birds in human settlements is birdfeeders. At a large spatial scale, we experimentally provided birdfeeders with four different kinds of food to analyze exploitation and use of a novel food supply provided by humans. Nine species started foraging at the new birdfeeders. The species that exploited the new feeders the fastest was the great tit. Use of novel food sources was faster in urban habitats and the presence of other feeders reduced the time until a new feeder was located. Urbanization may be associated with behavioural skills, technical innovations and neophilia resulting in faster discovery of new food sources. This process is accelerated by the experience of feeder use in the vicinity, with a strong modifying effect of the number of domestic cats.

Food availability is an important environmental cue when animals decide how much to invest in maintenance and reproduction, ultimately affecting population size^1^,^2^. In seasonal environments, winter is a critical period for survival for many bird species^3^,^4^,^5^,^6^. Therefore, adaptations that allow discovery and use of ephemeral food should be favoured by natural selection^7^,^8^. Thus the ability to develop novel behaviour innovations should facilitate exploitation of novel food resources and hence increase the prospects of survival in the new environment^9^,^10^. Birdfeeders have increased in numbers in recent decades, and now constitute a massive food source in many parts of the temperate zone. They are directly associated with human settlements across a range of surrounding habitats varying from rural to highly urbanized^2^,^9^,^11^. In fact, many bird species are able to exploit this novel food source in urban and suburban environments, where they thrive in close proximity of humans^12^,^13^,^14^.

For economic, social and biodiversity reasons people often change the presence and the abundance of artificial feeders during winter^15^. In winter when the time budget of especially small birds is very tight, opportunities to find a rich new food source can provide a large advantage in terms of intra- and interspecific competition and hence survival prospects^15^,^16^. Therefore, exploratory behaviour should be an adaptive trait especially in situations of limiting resources and starvation^8^. On the other hand, dispersal skills of small passerines, both at a local and a regional scale, and the search for novel food is reduced by both intra- and interspecific competition^17^. However, predation is a stronger selective agent than competition affecting winter mortality of small birds^18^,^19^,^20^,^21^. The effects of presence of predators or cues left by predators may modify the use of foraging and roosting sites in winter^22^,^23^,^24^.

In summary, discovery of novel food resources may be important for survival, and as a consequence it will promote individual traits linked with personality, enabling use of novel food sources^8^. The ability to find and explore novel food sources provided by humans would be important for adaptation to novel habitats, and for understanding urbanization processes of animals^9^,^25^.

To the best of our knowledge, there is a lack of tests of how fast wintering birds recognize novel food sources, especially those provided in birdfeeders. Such studies have general implications, because winter bird feeding is one of the most widespread direct interactions between humans and nature with important social and environmental consequences^2^,^26^,^27^. Exploitation of such novel feeding sites may allow tests of differences in feeder exploitation between rural and urban habitats^2^,^28^. We conducted a manipulative experiment with birdfeeder locations recording the species and the sex at the start of use of novel food sources^29^. New birdfeeders should be visited faster in urban than in rural habitats because population density of birds is higher and hence intraspecific competition for resources more intense. Moreover, discovery behaviour may be modified by risk of predation^30^. Finally, discovery behaviour may depend on prior experience and hence the number of birdfeeders provided by humans if prior exposure to feeders causes a phenotypically plastic reduction in neophobia.

## Results

**Figure 1:**
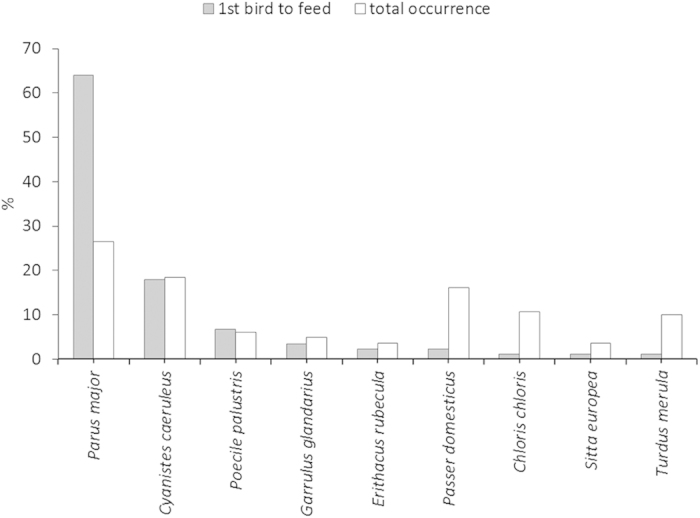
Abundance and frequency of first birds visiting a novel food source for different bird species. Sample sizes were 89 and 467 individuals, respectively, for the first bird and for the entire wintering bird community.

The wintering bird community around experimental birdfeeders contained 925 individuals belonging to 43 species (6.7, SD = 2.88; range 1–13). In 89 cases (64.5% of all 138 trials), a total of nine species visited experimental birdfeeders during 120 minutes and started to forage (Fig. 1). The proportion of species that discovered new food sources differed significantly from the species composition in the wintering bird community (Fig. 1; χ^2^ = 53.33, df = 8, P < 0.001). The bird species most frequently using the new food source was the great tit (64.0% of all cases). There were no significant differences between the frequencies of bird species first using the new food source in rural and urban environments (χ^2^ = 4.21, df = 3, P = 0.24). Male great tits were the most frequent users of new feeders (80%, χ^2^ = 4.14, df = 1, P = 0.04), despite an even sex ratio of this species in the study area (sex ratio: 92 females, 106 males; P = 0.39).

**Figure 2:**
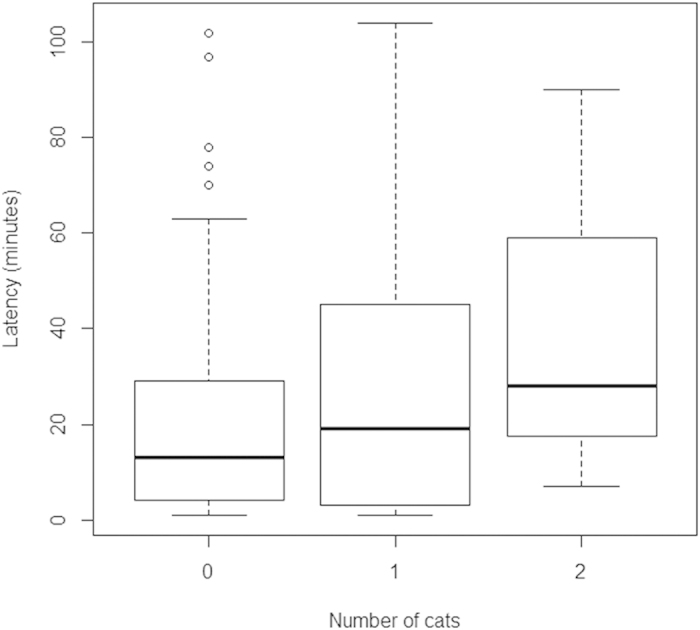
Differences in average time taken for birds to use a novel source of food (expressed in minutes) on y-axis, related to the presence and number of cats (x-axis) in the study area.

For all species in GAMM models, the urban environment was correlated with a decrease in time spent using the new source of food. The number of bird feeding stations and species richness of other birds that were seedeaters was positively correlated with the speed of use of the new food source, while the presence of cats was a cause of slower use of feeders (Table 1, Fig. 2).

The total occurrence of different bird species at feeders was strongly positively correlated with the frequency of technical innovations (log-transformed data, F = 14.03, df = 1, 6, r^2^ = 0.70, P = 0.0096, estimate (SE) = 0.669 (0.178)) after adjusting for body mass (log-transformed: F = 5.19, df = 1, 6, r^2^ = 0.46, P = 0.063, estimate (SE) = −0.439 (0.193)). This suggests that there is a cognitive basis for exploitation of bird feeders linked to the frequency of technical innovations^31^.

## Discussion

Faster detection and exploitation of novel food sources was recorded in urban habitat and increased by the number birdfeeders already present. Additionally, the presence of domestic cats negatively affected time required for discovery of new birdfeeders, suggesting that detection of novel food sources is affected by the risk of predation when searching for food. Finally, in the majority of cases the first species that discovered the novel food was the great tit, especially males, in a proportion larger than predicted from its abundance in the wintering bird community. The great tit is recognized across Europe as a species that often and regularly uses birdfeeders^32^, although varying among years and seasons, and even individually^33^. Therefore, great tits were used in many experiments on use of artificial food sources and the spread of social information^34^,^35^. Here we have shown that this species is not only a numerous visitor at feeders, but also the first to discover novel resources in the majority of cases. Male great tits generally take greater risks than females^36^, explaining why males were disproportionately frequent as the discoverers of novel food sources. Latency of some species is also potentially explained by the spectrum of diet, and therefore less attractiveness of novel food provided by humans^11^.We have shown that early exploitation of bird feeders is peculiar to specific species and even sexes. Indeed the frequency of occurrence at feeders was strongly predicted by a high frequency of technical innovations for a given body size. Because innovations by birds are more common in species with a relatively large brain size, we suggest that the abundance of birds frequenting novel feeders indirectly has an underlying neural basis^8^,^31^.

One the most interesting results were the differences between urban and rural habitats in time required to find novel food sources. Such differences can be the result of bird density and feeder density as already known food sources, but also skills of particular individual in exploratory behavior^8^. Food is not presented in exploration trials, so the motivation is assumed to be information gathering^16^,^37^. The costs of exploration may be the time, energy and attention diverted from other activities or risks, or indeed the potential unknown dangers of the novel object or the environment itself. We found empirical support for this suggestion because the latency until detection of novel food increased in the presence of a larger number of predators, domestic cats. The number of cats in human settlements is increasing, and they have a disproportionately large negative impact on biodiversity^38^ that accounts for a large fraction of mortality in wildlife including birds^39^. Urban habitats can support more risky and exploratory individuals than rural habitats^9^,^13^,^24^,^40^ because humans provide refuges against predators that generally avoid human proximity^41^ Species that readily taste new foods and/or develop novel foraging techniques are more likely to survive and to reproduce in a novel environment than individuals belonging to a more stereotyped species with less exploratory behavior^10^. We did not find a significant effect of temperature on discovery of birdfeeders as in other studies^42^, although the effect of weather is probably more important in the use of already known places than when searching for new sites^15^,^16^,^43^.

Two broad conclusions can be drawn from our field experiments. First, exploratory behaviour and search for novel food is influenced by multiple sources of variation such as rural vs. urban habitats, the presence of other birdfeeders provided earlier, and the presence of domestic cats. Second, there is significant intra- and interspecific variation in search for novel food sources, and part of this variation is due to sex, and an ability to innovate by particular bird species. This implies that a range of different factors contribute to recruitment of birds to novel sources of food and hence for the diversity of the winter bird community in urban environments.

## Methods

### Field methods

Data were collected during December 2013-February 2014 in eight cities and nearby rural areas across Poland (for more details on cities and map of location, see ). In total 138 experimental trials (80 and 58 in rural and urban areas, respectively) were carried out during days (1–4 hrs after sunrise) with favourable weather conditions (no snow or rain, strong wind). The site of the experiment was chosen randomly. To attract as many species as possible, in each trial one birdfeeder contained at the bottom four different trays (changed randomly for each trial) with four different kinds of food (contain carbohydrates and lipids): animal fat, dry fruits of rowanberry, sunflowers and millet seeds. Birdfeeders of a single model for all trials were used across the entire country, each being in the shape of a small house with a roof, and a 1.20 m pole that was dug into the ground (grass/soil) and was transported by car to the site of the experiment.

Before starting observations of activity at the birdfeeder, to quantify the composition of the local wintering bird community we recorded birds at three points at distances of 100 m from the feeder, located at virtual triangle tops with the birdfeeder in the middle, birds were counted using the point-counting method with 5-minute observations at each point. Data from point-counts were summarized and used to describe the winter bird community around feeders. Additionally at the start of the experiment temperature was also noted, and during the first 15 minutes of feeder watching we recorded the number of cats and dogs, as well as human walkers within a distance of 100 m (Table 2). At a radius of 100 m from experimental birdfeeders we also recorded all other feeders provided by humans because they potentially attract birds in the vicinity. Experimental birdfeeders were provided at a distance larger than 50 m from already existing (i.e. non-experimental) birdfeeders provided by humans.

When the new experimental birdfeeder was provided at a specific site, it was observed for 120 minutes from a distance (e. g. from a parked car with good visibility) noting when and which bird species first started to explore food in the feeder, and latency time (minutes since the start of the experiment) of this first bird was noted. Immediately, at least after 10 min when the first bid used the experimental birdfeeder, or if no birds arrived at the birdfeeders following an experimental trial of 120 min, the experiment was terminated and the observer with the experimental feeder moved to another place, located at least 2 km from the previous one. All sampled sites, studied in the proximity of the eight Polish cities, were classified as ‘urban’ or ‘rural’ following the main landscape. During observations birds were sexed from a distance with binoculars, although sufficient data on sex was only collected for the great tit *Parus major*. Bird species richness was calculated as the sum of all seed-eating bird species recorded in each sampled site.

The methods were carried out in accordance with the approved guidelines and Polish national law. Moreover, because is not really experimental study using animals, but only observations from some distance, additional approval by the local ethical committee was not required.

### Statistics

The sampled sites were treated as statistically independent observations because the values of spatial autocorrelation were low (Mantel test p > 0.05, n = 89)^44^,^45^. A Mantel test measures the correlation between two matrices typically containing measures of distance. The Mantel test was performed using the package ‘ade4’ for R package.

Occurrences of the most frequent bird species first using the new food source was analyzed in terms of differences among habitats (urban or rural) and difference in sex ratio of the great tit using new feeders with a chi-square test^46^,^47^, and the Bonferroni adjustment method was applied, multiplying the p-values with the number of comparisons. Differences in average time taken for birds to use a novel source of food (expressed in minutes) between male and female great tits were tested with the Mann-Whitney U-test.

Finally, a generalized additive mixed model (GAMM) with the package ‘mgcv’^7^,^48^ was used to study differences in latency when the first bird started to use the novel food source. GAMMs are especially designed for inference of relationships of clustered and correlated data by adding random effects to the additive predictor, which account for that correlation. In this study, GAMM was used to take into account any potential effect on the studied behaviour, related to differences among cities where data were collected.

The variable “minutes” was used as response variable. The covariates entered in the full model were bird species richness, date, temperature, environment (urban or rural), number of dogs, cats and human walkers, number of feeders and number of corvids. In this case, we specified as random effects the grouping structure of the data defined by the “city identity” where the observations were collected. A Poisson distribution error was assumed for the response variable.

The frequency of technical innovations was estimated as the sum of novel feeding techniques, novel techniques in an anthropogenic context, novel parasitic behaviour, novel commensal behaviour, novel mutualistic behaviour, novel prototool behaviour, novel tool behaviour and novel caching behaviour^36^.

## Additional Information

**How to cite this article**: Tryjanowski, P. *et al*. Who started first? Bird species visiting novel birdfeeders. *Sci. Rep*. **5**, 11858; doi: 10.1038/srep11858 (2015).

## Figures and Tables

**Table 1:** Differences in time when the first bird arrived at a new feeder in relation to environment, temperature, number of cats, dogs, humans, corvids, bird species richness and number of feeders. Results of GAMM: R-sq. (adj) = 0.295, scale est = 10.588, n = 89, df = 9. Random effects (SD): intercept = 0.513, residual = 3.254.

Variable	Estimate	SE	*t*	*P*
Intercept	4.277	0.368	11.614	<0.001
Environment (urban)	−0.856	0.231	−3.699	0 .001
Temperature	0.004	0.019	0.234	0.816
No. cats	0.386	0.171	2.256	0.027
No. dogs	0.156	0.134	1.159	0.250
No. human walkers	−0.001	0.046	−0.022	0.983
No. feeders	−0.189	0.063	−3.011	0.003
Bird species richness	−0.098	0.051	−1.928	0.057
No. corvids	0.112	0.137	0.816	0.417

**Table 2:** Characteristic of explanatory variables. Data are presented as means ± SE.

Variable	Urban n = 58	Rural n = 80
No. cats	0.38 ± 0.08	0.15 ± 0.04
No. dogs	0.81 ± 0.14	0.25 ± 0.06
No. human walkers	8.84 ± 2.41	0.84 ± 0.19
No. feeders	0.41 ± 0.08	2.03 ± 0.28

## References

[b1] Newton I. Population limitation in birds (Academic Press, London, 1998).

[b2] Robb G. N., McDonald R. A., Chamberlain D. E. & Bearhop S. Food for thought: Supplementary feeding as a driver of ecological change in avian populations. Front. Ecol. Environ. 6, 476–484 (2008).

[b3] Houston A. I. & McNamara J. M. A theoretical investigation of the fat reserves and mortality levels of small birds in winter. Ornis Scand. 24, 205–219 (1993).

[b4] Bednekoff P. A. & Krebs J. R. Great tit fat reserves, effects of changing and unpredictable feeding day length. Funct. Ecol. 9, 457–462 (1995).

[b5] Pravosudov V. V. & Grubb T. C. Jr. Energy management in passerine birds during the nonbreeding season: A review. Curr. Ornithol. 14, 189–234 (1997).

[b6] Atkinson P. W., Fuller R. A. & Vickery J. A. Large-scale patterns of summer and winter bird distribution in relation to farmland type in England and Wales. Ecography 25, 466–480 (2002).

[b7] Gosling S. D. From mice to men, what can we learn about personality from animal research. Psychol. Bull. 127, 45–86 (2000). 11271756 10.1037/0033-2909.127.1.45

[b8] Ducatez S., Clavel J. & Lefebvre L. Ecological generalism and behavioral innovation in birds. Technical intelligence or the simple incorporation of new foods? J. Anim. Ecol. 84, 79–89 (2015). 10.1111/1365-2656.12255 24910268

[b9] Møller A. P. Successful city dwellers, a comparative study of the ecological characteristics of urban birds in the Western Palearctic. Oecologia 159, 849–858 (2009). 19139922 10.1007/s00442-008-1259-8

[b10] Sol D., Griffin A. S., Bartomeus I. & Boyce H. Exploring or Avoiding Novel Food Resources? The Novelty Conflict in an Invasive Bird. PLoS ONE 6, e195352011 (2011). 10.1371/journal.pone.0019535 PMC3097186 21611168

[b11] Galbraith J. A. . Risks and drivers of wild bird feeding in urban areas of New Zealand. Biol. Conserv. 180, 64–74 (2014).

[b12] Luniak M. Synurbization – adaptation of animal wildlife to urban development. Pp. 50–55. In Shaw W. W., Harris L. K. & Vandruff L. (Eds.), Proceedings of the 4th International Urban Wildlife Symposium. University of Arizona, Tucson, Arizona, U.S.A. (2004).

[b13] Ditchkoff S. S., Saalfeld S. T. & Gibson C. J. Animal behavior in urban ecosystems: Modifications due to human-induced stress. Urban Ecosyst. 9, 5–12 (2006).

[b14] Fuller R. A., Irvine, K. N., Davies, Z. G., Armsworth, P. R. & Gaston K. J. Interactions between people and birds in urban landscapes. Stud. Avian Biol. 45, 249–266 (2012).

[b15] Maciusik B., Lenda M. & Skórka P. Corridors, local food resources and climatic conditions affect the utilization of the urban environment by the Black-headed Gull *Larus ridibundus* in winter. Ecol. Res. 25, 263–272 (2010).

[b16] Tvardikova K. & Fuchs R. Tits recognize the potential dangers of predators and harmless birds in feeder experiments. J. Ethol. 30, 157–165 (2012).

[b17] Dhondt A. A. & Eyckerman R. Competition between the great tit and the blue tit outside the breeding season in field experiments. Ecology 61, 1291–1296 (1980).

[b18] Ekman J. Tree use and predator vulnerability of wintering passerines. Ornis Scand. 17, 261–267 (1986).

[b19] Carrascall M. & Moreno E. Proximal costs and benefits of heterospecific social foraging in the Great Tit, Parus major. Can. J. Zool. 70, 1947–1952 (1992).

[b20] Suhonen J. Predation risk influences the use of foraging sites by tits. Ecology 74, 1197–1203 (1993).

[b21] Kullberg C. Spatial niche dynamics under predation risk in the willow tit *Parus montanus*. J. Avian Biol. 29, 235–240 (1998).

[b22] Lima S. L. Nonlethal effects in the ecology of predator-prey interaction. What are the ecological effects of anti-predator decision-making? BioScience 48, 25–34 (1998).

[b23] Ekner A. & Tryjanowski P. Do small hole nesting passerines detect cues left by a predator? A test on winter roosting sites. Acta Ornithol. 43, 107–111 (2008).

[b24] Seress G., Bókony V., Heszberger J. & Liker A. Response to predation risk in urban and rural house sparrows. Ethology 117, 896–907 (2011).

[b25] Jokimäki J., Suhonen J., Jokimäki-Kaisanlahti M. L. & Carbó-Ramírez P. Effects of urbanization on breeding birds in European towns, Impacts of species traits. Urban Ecosyst. 10.1007/s11252-014-0423-7 (2014).

[b26] Tratalos J., Fuller R. A., Warren P. H., Davies R. G. & Gaston K. J. Urban form, biodiversity potential and ecosystem services. Landscape Urban Plan. 83, 308–317 (2007).

[b27] Horn D. J. & Johansen S. M. A. comparison of birdfeeding practices in the United States and Canada. Wildlife Soc. Bull. 37, 293–300 (2013).

[b28] Horn D. J., Fairbairn S. E. & Hollis R. J. Factors influencing the occurrence of birds that use feeders in Iowa. J. Iowa Acad. Sci. 109, 8–18 (2002).

[b29] Amrhein V. Wild bird feeding (probably) affects avian urban ecology. In: Gil D. & Brumm H. Avian Urban Ecology, Behavioural and Physiological Adaptations (Oxford University Press, 2014).

[b30] Lima S. L. Predators and the breeding bird: behavioral and reproductive flexibility under the risk of predation. Biol. Rev. 84, 485–513 (2009). 19659887 10.1111/j.1469-185X.2009.00085.x

[b31] Overington S. E., Bogert N. J., Morand-Ferron J. & Lefebvre L. Technical innovation drive the relationship between innovativeness and residual brain size in birds. Anim. Behav. 78, 1001–1010 (2009).

[b32] Chamberlain D. E. . Annual and seasonal trends in the use of garden feeders by birds in winter. Ibis 147, 563–575 (2005).

[b33] Dingemanse N. J. . Variation in personality and behavioural plasticity across four populations of the great tit *Parus major*. J. Anim. Ecol. 81, 116–126 (2012). 21692798 10.1111/j.1365-2656.2011.01877.x

[b34] Suhonen J. Predation risk influences the use of foraging sites by tits. Ecology 74, 1197–1203 (1993).

[b35] Farine D. R. & Lang S. D. The early bird gets the worm, foraging strategies of wild songbirds lead to the early discovery of food sources. Biol. Lett. 9, 10.1098/rsbl.2013.0578 (2013). PMC3871345 24108676

[b36] Regelmann K. & Curio E. Why do great tit (*Parus major*) males defend their brood more than females do? Anim. Behav. 34, 1206–1214 (1986).

[b37] Mettke-Hofmann C., Winkler H. & Leisler B. The significance of ecological factors for exploration and neophobia in parrots. Ethology 108, 249–272 (2002).

[b38] Baker P. J. . Cats about town: is predation by free‐ranging pet cats *Felis catus* likely to affect urban bird populations? Ibis 150, 86–99 (2008).

[b39] Loss S. R., Will T. & Marra P. P. The impact of free-ranging domestic cats on wildlife of the United States. Nature Commun. 4, 1396. 10.1038/ncomms2380 (2013). 23360987

[b40] Maklakov A. A. . Brains and the city, big-brained passerine birds succeed in urban environments. Biol. Lett. 7, 730. 10.1098/rsbl.2011.0341 (2011). 21525053 PMC3169078

[b41] Møller A. P. Urban areas as refuges from predators and flight distance of prey. Behav. Ecol. 23, 1030–1035 (2012).

[b42] Wilson W. E. Jr. The effects of supplemental feeding on wintering Black-capped Chickadees (*Poecile atricapilla*) in central Maine, Population and individual responses. Wilson Bull. 113, 65–72 (2001).

[b43] McNamara J. R., Houston A. & Lima S. Foraging routines of small birds in winter, A theoretical investigation. J. Avian Biol. 25, 287–302 (1994).

[b44] Tryjanowski P. . Winter bird assemblages in rural and urban habitats, a national survey. PLoS One, resubmitted (2015). 10.1371/journal.pone.0130299 PMC4472663 26086819

[b45] Manly B. F. J. Randomization, Bootstrap and Monte Carlo Methods in Biology. Third Edition (Chapman and Hall/CRC, Boca Raton, 2006).

[b46] Sokal R. R. & Rohlf F. J. Biometry, the principles and practice of statistics in biological research (W.H. Freeman, New York, NY, 1995).

[b47] Wood S. N. *Generalized Additive Models. An Introduction with R* (London, UK, Chapman and Hall/CRC, Boca Raton, 2006).

[b48] Wood S. N. Stable and efficient multiple smoothing parameter estimation for generalized additive models. J. Am. Stat. Assoc. 99, 673–686 (2004).

